# Platelet activation in breast cancer: mechanisms and therapeutic opportunities – a narrative review

**DOI:** 10.1097/MS9.0000000000003091

**Published:** 2025-05-21

**Authors:** Emmanuel Ifeanyi Obeagu

**Affiliations:** Department of Biomedical and Laboratory Science, Africa University, Mutare, Zimbabwe

**Keywords:** breast cancer, coagulation, platelet activation, therapeutic strategies, tumor metastasis

## Abstract

Platelet activation is increasingly recognized as a key factor in breast cancer progression, contributing to tumor growth, metastasis, and immune modulation. Platelets interact with tumor cells to promote their survival, facilitate metastasis, and enhance the formation of a pro-inflammatory microenvironment. These interactions are mediated by adhesion molecules such as P-selectin and glycoprotein receptors on both platelets and tumor cells. Additionally, activated platelets release growth factors like vascular endothelial growth factor and platelet-derived growth factor, which promote angiogenesis and tumor vascularization, crucial steps in cancer progression. The coagulation cascade, triggered by platelet activation, further enhances tumor cell dissemination and metastasis. Elevated levels of procoagulant activity in platelets, particularly through the expression of tissue factor, lead to increased thrombin generation, facilitating the formation of fibrin-rich clots that protect circulating tumor cells from immune surveillance. Platelet-derived factors also exert immunomodulatory effects, helping cancer cells evade detection by the immune system, thereby supporting tumor growth and spread.

## Introduction

Breast cancer is one of the most prevalent malignancies globally, affecting millions of individuals each year. It remains a leading cause of cancer-related death, largely due to metastasis, which occurs when cancer cells spread from the primary tumor site to distant organs. The complex and multifactorial nature of breast cancer progression involves intricate interactions between tumor cells, the extracellular matrix, blood vessels, and immune cells. Among these factors, platelet activation has emerged as a critical player in cancer progression, facilitating tumor growth, metastasis, and immune evasion. While platelets are traditionally known for their role in coagulation, recent research highlights their significant contribution to cancer biology, including in breast cancer^[1,2]^. Platelets are small, anucleate cell fragments that circulate in the blood and are primarily involved in blood clotting. However, beyond their hemostatic function, platelets interact with various tumor cells and influence the progression of cancer. Platelet activation occurs when platelets encounter certain stimuli, such as exposure to collagen or thrombin, leading to a cascade of intracellular signaling events. These events cause platelets to release a variety of bioactive molecules, including growth factors, cytokines, and pro-coagulant proteins. These molecules not only facilitate platelet aggregation but also influence the tumor microenvironment (TME) and promote cancer progression^[3,4]^. One of the most critical roles of platelets in cancer is their ability to promote tumor cell survival and enhance their metastatic potential. Platelets shield circulating tumor cells (CTCs) from immune detection, increasing their ability to establish secondary tumors in distant organs. Tumor cells, in turn, secrete signals that activate platelets, creating a positive feedback loop. Platelets also facilitate the adhesion of tumor cells to endothelial cells in blood vessels, a crucial step in the metastatic spread of breast cancer. By forming platelet–tumor cell aggregates, platelets help tumor cells evade immune surveillance and establish metastatic lesions in distant tissues^[5]^.

**Highlights**

- Platelet activation promotes breast cancer progression by enhancing tumor cell growth, invasion, and metastasis.
- Activated platelets release growth factors like VEGF, supporting angiogenesis in breast tumors.
- Platelet–tumor interactions facilitate immune evasion by shielding cancer cells from immune detection.
- Targeting platelet activation pathways (e.g., COX-2 inhibitors, antiplatelet drugs) offers therapeutic potential in breast cancer management.
- Platelet-derived microparticles contribute to chemoresistance and tumor microenvironment remodeling.

In addition to promoting tumor metastasis, platelet activation plays a central role in angiogenesis, the process by which new blood vessels form to supply growing tumors with nutrients and oxygen. Platelets release vascular endothelial growth factor (VEGF) and platelet-derived growth factor (PDGF), both potent angiogenic factors that stimulate the proliferation and migration of endothelial cells. These growth factors promote the development of a new vascular network within tumors, ensuring their continued growth and survival. By contributing to angiogenesis, platelets provide the tumor with the necessary infrastructure to expand and metastasize^[6,7]^. Another critical mechanism through which platelets influence cancer progression is through their pro-coagulant activity. Activated platelets release tissue factor (TF), a protein that initiates the extrinsic coagulation cascade by activating factor VII. This leads to the production of thrombin, which further activates platelets and promotes fibrinogen conversion to fibrin, ultimately forming a clot. The fibrin-rich clot acts as a physical barrier that protects CTCs from immune attack, facilitating their survival in the bloodstream. This clot formation also creates a favorable microenvironment for tumor cells to invade tissues and establish secondary growths^[8,9]^. Platelets not only support tumor progression through direct interactions with tumor cells but also influence the surrounding stromal and immune cells. For example, platelets release cytokines such as transforming growth factor-beta (TGF-β), which can alter the immune landscape of the TME. TGF-β can suppress anti-tumor immune responses, further promoting tumor growth and metastasis. Additionally, platelets modulate the activity of immune cells, including macrophages, dendritic cells, and T cells, to help tumor cells evade immune detection and destruction^[10,11]^.

## Aim

The aim of this review is to explore the mechanisms of platelet activation in breast cancer and evaluate the therapeutic opportunities that target platelet function as a strategy for limiting tumor progression and metastasis.

## Justifications of the review

Platelet activation is increasingly recognized as a crucial factor in the metastatic spread and progression of breast cancer. Platelets not only facilitate the survival of CTCs but also support tumor angiogenesis and immune evasion. Understanding the specific molecular mechanisms through which platelets contribute to these processes provides a compelling justification for this review. Given the significance of platelet-mediated processes in cancer progression, exploring therapeutic strategies to disrupt these interactions could offer new avenues for managing advanced-stage breast cancer, where metastasis is the leading cause of mortality^[12,13]^. Despite significant advancements in cancer treatment, metastatic breast cancer remains a major challenge due to its aggressive nature and resistance to conventional therapies. Platelet-targeted therapies, including anti-platelet agents and inhibitors of platelet–tumor cell interactions, are emerging as potential therapeutic strategies. This review justifies the need to explore these novel approaches, as existing therapies often fail to adequately address the role of platelets in metastasis. By focusing on the potential of targeting platelet activation, this review can highlight innovative treatments that may complement or enhance current breast cancer therapies, offering more effective solutions for managing metastasis and improving patient outcomes^[14,15]^. The clinical implications of platelet activation in breast cancer are significant, particularly in patients with advanced disease. Thrombosis is a common complication in cancer patients, and its association with breast cancer metastasis underscores the importance of platelet activity in disease progression. Current treatments primarily focus on tumor targeting and immune modulation, but platelets continue to facilitate cancer spread, warranting a comprehensive review of strategies to inhibit platelet activation. Given the unmet need for effective treatments that target metastatic mechanisms beyond traditional chemotherapy, this review serves to inform the development of new therapeutic options for clinical use, potentially improving survival and quality of life for breast cancer patients^[16,17]^.

The mechanistic insights provided by this review will contribute to the growing body of knowledge on the interplay between platelets and tumor biology. With the increasing recognition of platelets as active participants in cancer metastasis, this review justifies the need for further research in this area to identify new biomarkers for early detection and assess the impact of platelet-targeted therapies on patient outcomes. By synthesizing current literature on platelet activation in breast cancer, this review aims to bridge the gap between basic science and clinical practice, offering insights that could lead to the development of more targeted and personalized therapeutic strategies^[18]^. The justification for this review is further supported by the potential for combining platelet-targeted therapies with existing treatments such as chemotherapy, immunotherapy, and radiation. Platelet inhibition could improve the efficacy of these therapies by reducing tumor cell survival, metastasis, and immune suppression. Understanding how platelet-targeted interventions interact with other treatment modalities is crucial for developing combination therapies that are more effective in tackling metastatic breast cancer. This review, therefore, aims to provide a comprehensive overview of how targeting platelet activation can enhance therapeutic outcomes when used in conjunction with other treatment approaches.

## Review methods

### Literature search strategy

The review process involved a comprehensive search of relevant literature on the role of platelet activation in breast cancer and therapeutic strategies aimed at targeting platelet function. A systematic search was conducted across multiple databases, including PubMed, Scopus, Web of Science, and Google Scholar. Keywords such as “platelet activation in breast cancer,” “platelet-tumor interactions,” “platelet-targeted therapy,” “thrombosis in cancer,” and “metastasis and platelets” were used to identify peer-reviewed articles, clinical trials, and preclinical studies published. The search was restricted to articles in English, and relevant publications were selected based on their alignment with the review objectives. Articles were assessed for quality using criteria such as study design, sample size, and relevance to the review topic. Abstracts and full-text articles were screened for their contribution to understanding the mechanisms of platelet activation in breast cancer and potential therapeutic approaches.

**Inclusion and Exclusion Criteria**: Studies were included in the review if they met the following criteria:

Focused on the role of platelet activation in breast cancer progression, metastasis, or tumor biology.

- Discussed therapeutic strategies aimed at inhibiting platelet activation, including preclinical and clinical studies.
- Addressed molecular mechanisms of platelet–tumor cell interactions, platelet-mediated immune modulation, and tumor angiogenesis.

Provided data on the impact of platelet-targeted therapies on breast cancer outcomes.

Studies were excluded if they:

Focused on platelet activation in non-cancerous diseases or other types of cancer.

- Did not contribute substantial data on the mechanisms of platelet activation or therapeutic strategies in breast cancer.

Were not published in peer-reviewed journals or lacked scientific rigor.

### Platelet activation mechanisms in breast cancer

Platelet activation in breast cancer is a complex process that involves the interaction between platelets and tumor cells, as well as the surrounding TME. This interaction contributes significantly to tumor progression, metastasis, and immune evasion. Several mechanisms are involved in platelet activation, and these mechanisms play a critical role in facilitating tumor growth and the spread of cancer to distant organs. Below, we explore the primary mechanisms by which platelets are activated in breast cancer^[19]^.

1. **Platelet–Tumor Cell Interactions**: Platelet activation begins with direct interactions between platelets and tumor cells. Tumor cells, through the release of signaling molecules, can bind to platelets and trigger their activation. This is primarily mediated by adhesion molecules such as P-selectin on platelets and integrins, such as *α*IIbβ3, on both platelets and tumor cells. P-selectin on activated platelets binds to P-selectin glycoprotein ligand-1 (PSGL-1) on tumor cells, promoting the formation of platelet–tumor cell aggregates. These aggregates help protect tumor cells from immune detection and promote their survival in the bloodstream, ultimately aiding in the metastatic spread of cancer^[12]^.
2. **Release of Bioactive Molecules**: Once activated, platelets release a range of bioactive molecules that influence cancer biology. Platelets secrete growth factors such as VEGF, PDGF, and fibroblast growth factors (FGFs). These molecules promote angiogenesis, a crucial process for tumor growth, by stimulating the proliferation and migration of endothelial cells to form new blood vessels. The increased blood supply ensures that the tumor receives adequate oxygen and nutrients, further promoting tumor growth and metastasis. Additionally, platelets release pro-inflammatory cytokines, such as interleukins (IL-1β, IL-6), that contribute to the inflammatory environment within the tumor, enhancing its progression^[20]^.
3. **Procoagulant Activity and Tumor Metastasis**: Activated platelets also exhibit enhanced procoagulant activity, which plays a pivotal role in promoting metastasis. Platelets express TF on their surface, a key initiator of the extrinsic coagulation cascade. TF binds to factor VIIa, activating thrombin, which in turn induces platelet aggregation and clot formation. This thrombin-induced clotting process forms a fibrin-rich network around CTCs, protecting them from shear forces and immune cell-mediated elimination. The clot acts as a physical scaffold, aiding in the establishment of metastatic lesions in distant organs. This process, known as “platelet aggregation-induced metastasis,” facilitates the survival of CTCs during their journey through the bloodstream and their subsequent colonization of secondary sites^[21]^.
4. **Platelet-Mediated Immune Modulation**: Platelets can modulate the immune response within the TME, influencing cancer progression and immune evasion. Upon activation, platelets release cytokines such as TGF-β, which suppress immune responses, particularly the activity of cytotoxic T lymphocytes and natural killer (NK) cells. TGF-β promotes the differentiation of regulatory T cells (Tregs), which further dampen the anti-tumor immune response. Platelets also interact with other immune cells, such as macrophages and dendritic cells, promoting a pro-tumor inflammatory environment that supports tumor growth and immune escape. This immune modulation by platelets contributes significantly to the ability of breast cancer cells to evade immune surveillance and establish metastatic growth^[15]^.
5. **Role of Thrombin in Platelet Activation**: Thrombin, a central enzyme in the coagulation cascade, plays a pivotal role in platelet activation in breast cancer. Thrombin is generated during platelet activation and induces further platelet aggregation and secretion of pro-inflammatory and angiogenic factors. Thrombin interacts with protease-activated receptors (PARs) on platelets, leading to their activation and secretion of additional molecules such as VEGF and PDGF. Thrombin also promotes the conversion of fibrinogen to fibrin, leading to clot formation, which, as mentioned earlier, protects CTCs in circulation and supports metastatic dissemination. The thrombin-mediated activation of platelets is thus a key mechanism through which platelets contribute to tumor progression and metastasis^[22]^.
6. **Adhesion Molecules and Platelet–Tumor Cell Aggregation**: Adhesion molecules are crucial for platelet activation and their subsequent interaction with tumor cells. One of the primary adhesion molecules involved in platelet activation is P-selectin, which is expressed on the surface of activated platelets. P-selectin binds to PSGL-1 on the surface of tumor cells, leading to the formation of platelet–tumor cell aggregates. These aggregates play a significant role in protecting tumor cells from immune recognition and increasing their ability to survive in the bloodstream. Integrins such as αIIbβ3 and α2β1 are also involved in platelet adhesion to tumor cells, facilitating the formation of stable platelet–tumor cell aggregates that promote tumor cell dissemination and metastasis^[23]^.
7. **Microvesicles and Platelet Activation**: Platelets also release microvesicles or microparticles upon activation, which can further promote cancer progression. These microvesicles are small membrane-bound particles that contain various bioactive molecules, including proteins, RNA, and lipids. Microvesicles derived from platelets can interact with tumor cells and endothelial cells, promoting angiogenesis and increasing the metastatic potential of the tumor. These microvesicles may also play a role in altering the TME by modulating immune cell activity, promoting tumor cell survival, and enhancing resistance to chemotherapy^[24]^.
8. **Platelet Role in Tumor Angiogenesis**: In addition to releasing growth factors like VEGF and PDGF, platelets contribute to tumor angiogenesis through their ability to influence endothelial cell behavior. Activated platelets can promote the proliferation, migration, and tube formation of endothelial cells, all of which are essential for the formation of new blood vessels in the tumor. This angiogenic response helps to provide the growing tumor with the necessary blood supply to sustain its growth and support metastatic dissemination. The role of platelets in angiogenesis is particularly important in the context of breast cancer, where the formation of new blood vessels is critical for both primary tumor growth and the development of metastases^[25]^.
9. **Platelet Contributions to the TME**: Platelet activation significantly affects the TME, which consists of tumor cells, stromal cells, extracellular matrix components, and immune cells. The TME is a dynamic environment that promotes tumor progression and metastasis. Activated platelets contribute to this environment by releasing factors that promote a pro-inflammatory state, enhance angiogenesis, and increase the invasiveness of tumor cells. Platelets also contribute to the remodeling of the extracellular matrix (ECM), facilitating tumor cell migration and invasion. Through these various mechanisms, platelets play a key role in shaping the TME to support tumor growth and metastatic dissemination^[26]^.
10. **Platelet–Tumor Interaction in Bone Metastasis**: In breast cancer, metastasis to bone is a common and debilitating complication. Platelets play an essential role in the metastatic spread of tumor cells to bone tissue. Tumor cells can bind to activated platelets through specific receptors, which aids in their lodging within the bone marrow and bone vasculature. In addition, platelets secrete factors such as TGF-β, which can promote osteoclastogenesis, a process that leads to bone resorption. This bone resorption creates a favorable environment for the growth of metastatic breast cancer cells in bone tissue. Thus, platelet–tumor interactions not only facilitate the initial stages of bone metastasis but also contribute to the progression of bone lesions^[27]^.

### Therapeutic opportunities in targeting platelet activation in breast cancer

Targeting platelet activation presents a promising therapeutic approach for managing breast cancer, particularly in the context of metastasis, tumor progression, and immune evasion. Given that platelets are involved in numerous processes that promote cancer growth and dissemination, interventions designed to modulate platelet function could yield significant clinical benefits. Several strategies have emerged to block platelet activation, either by targeting the molecular mechanisms driving platelet–tumor cell interactions or by inhibiting the downstream effects of platelet activation, such as procoagulant activity and immune modulation. Below are some of the therapeutic opportunities in targeting platelet activation in breast cancer^[3]^.

1. **Anti-Platelet Agents**: One of the most well-established approaches for targeting platelet activation is the use of anti-platelet agents. These drugs, such as aspirin, clopidogrel, and ticagrelor, inhibit platelet aggregation by blocking the activation of key platelet receptors, such as cyclooxygenase-1 (COX-1) or P2Y12 receptors. Aspirin, for example, irreversibly inhibits COX-1, preventing the production of thromboxane A2, a potent platelet activator. These agents can disrupt the platelet–tumor cell interaction, reducing platelet aggregation around CTCs and limiting their ability to form metastatic lesions. Additionally, anti-platelet therapies may reduce the risk of thrombosis in cancer patients, which is commonly seen in breast cancer due to increased platelet activation. However, the efficacy of these agents in preventing breast cancer metastasis remains under investigation, with clinical trials showing mixed results^[28]^.
2. **Inhibition of Platelet Aggregation and Tumor Cell Adhesion**: Interfering with the adhesion between platelets and tumor cells is another promising strategy to reduce platelet-mediated metastasis. Drugs that block key adhesion molecules, such as P-selectin and integrins, could prevent platelet–tumor cell aggregates from forming. For instance, monoclonal antibodies targeting P-selectin (e.g., bimosiamose) have been shown to inhibit platelet–tumor cell interactions, thus reducing tumor cell survival in the bloodstream. Similarly, inhibitors of integrins, such as cilengitide, which block platelet aggregation by preventing integrin interactions, have shown potential in reducing tumor spread. These therapies may provide an effective means of preventing the seeding of tumor cells to distant organs, thereby limiting metastatic growth in breast cancer^[29]^.
3. **Thrombin Inhibitors and Coagulation Blockade**: Since thrombin plays a central role in platelet activation and tumor metastasis, inhibiting thrombin or its downstream effects represents a promising therapeutic approach. Direct thrombin inhibitors, such as dabigatran, or indirect inhibitors, such as heparin, have shown potential in disrupting the procoagulant environment within tumors. By inhibiting thrombin’s ability to activate platelets and generate fibrin clots around tumor cells, these agents could reduce the metastatic potential of CTCs. Clinical trials assessing the use of anticoagulants and thrombin inhibitors in cancer treatment have yielded mixed results, with some studies suggesting that these drugs may also reduce tumor growth and metastasis by disrupting the coagulation cascade^[30]^.
4. **Targeting PDGFs**: Activated platelets release various growth factors, such as VEGF, PDGF, and FGFs, which promote angiogenesis and tumor progression. Inhibiting the release or activity of these growth factors represents a potential therapeutic strategy. Anti-VEGF therapies, such as bevacizumab, have been tested in breast cancer and shown to reduce angiogenesis, limiting the blood supply to tumors. Additionally, blocking the PDGF receptor using small-molecule inhibitors could reduce platelet-driven angiogenesis and tumor growth. By targeting these pro-tumorigenic molecules, therapies could reduce the ability of platelets to support tumor vascularization and progression^[31]^.
5. **Platelet-Immune Interaction Modulation**: Platelets play an essential role in modulating the immune microenvironment within tumors, particularly in suppressing anti-tumor immunity. By releasing cytokines such as TGF-β, platelets promote immune evasion and tumor progression. Targeting platelet-mediated immune modulation could, therefore, enhance the immune system’s ability to detect and eliminate tumor cells. For instance, TGF-β inhibitors or monoclonal antibodies that block platelet-derived immune-modulatory signals may enhance the activity of cytotoxic T cells and NK cells, improving the anti-tumor immune response. Combining platelet-targeted therapies with immune checkpoint inhibitors, such as anti-PD-1 or anti-CTLA-4 antibodies, could lead to synergistic effects, boosting the immune system’s ability to fight breast cancer^[32]^.
6. **Targeting Platelet-Derived Microvesicles**: Platelet-derived microvesicles or microparticles are small particles released from activated platelets that carry bioactive molecules, including RNA, lipids, and proteins. These microvesicles have been implicated in tumor progression, angiogenesis, and metastasis. By blocking the release or activity of platelet-derived microvesicles, it may be possible to limit the ability of platelets to promote cancer growth. Therapies targeting the release of microvesicles or their interaction with tumor cells could reduce the metastatic potential of breast cancer. For example, the use of small molecules that inhibit microvesicle formation or neutralizing antibodies could help prevent platelet-induced cancer progression^[24]^.
7. **Anti-Cancer Platelet Inhibitors in Combination Therapy**: A promising therapeutic opportunity lies in the combination of platelet inhibitors with traditional cancer treatments, such as chemotherapy, radiation, and immunotherapy. By targeting platelet activation, these agents could potentiate the effects of other treatments. For example, platelet inhibitors may enhance the efficacy of chemotherapy by reducing tumor-induced thrombosis and limiting immune suppression. In combination with immunotherapies, platelet inhibitors could enhance immune cell infiltration and reduce the tumor’s ability to evade immune surveillance. Clinical trials exploring the combination of anti-platelet agents with chemotherapy and immunotherapy are ongoing and may provide new strategies for improving treatment outcomes in breast cancer patients^[33]^.
8. **Selective Targeting of Platelet Receptors**: Another avenue for therapeutic intervention is the selective targeting of platelet receptors involved in activation and platelet–tumor cell interactions. For example, antagonists that block the glycoprotein IIb/IIIa receptor, which is crucial for platelet aggregation, could reduce the ability of platelets to form aggregates with tumor cells. Similarly, targeting the PARs, which mediate thrombin-induced platelet activation, could inhibit platelet-driven pro-coagulant activity and metastasis. These targeted therapies could selectively block the pathways involved in platelet activation without causing widespread platelet dysfunction, thus minimizing bleeding risks^[34]^.
9. **Platelet Modulators and Nanoparticle-Based Therapies**: Nanotechnology offers an exciting approach for selectively targeting platelet activation in breast cancer. Nanoparticles can be engineered to deliver drugs specifically to activated platelets or tumor cells, improving the efficacy of platelet-targeted therapies. Nanoparticles loaded with anti-platelet agents, thrombin inhibitors, or anti-coagulants could selectively bind to platelet aggregates and tumor cells, providing localized treatment with minimal systemic side effects. Additionally, nanoparticles could be used in combination with imaging agents, allowing for better monitoring of platelet–tumor interactions and therapy outcomes in real time^[35]^.
10. **Gene Therapy and RNA-Based Approaches**: Gene therapy and RNA-based interventions represent cutting-edge strategies to target platelet activation in cancer. By delivering small interfering RNA or antisense oligonucleotides targeting key platelet activation molecules, such as P-selectin or integrins, it may be possible to prevent platelet–tumor cell interactions and reduce metastasis. Gene therapy approaches could also aim to modify the expression of pro-angiogenic or pro-inflammatory molecules in platelets, thereby limiting their ability to promote tumor growth and metastasis. Although still in early stages, these technologies hold significant promise for developing highly specific and targeted treatments for breast cancer.^[36-42]^

## Conclusion

Platelet activation plays a crucial role in the progression and metastasis of breast cancer, making it a significant target for therapeutic intervention. By promoting tumor cell survival, enhancing immune evasion, and facilitating metastasis through platelet–tumor cell interactions, platelets contribute to cancer spread and the development of thromboembolic complications in cancer patients. The mechanisms of platelet activation, including platelet aggregation, the release of growth factors, and interactions with tumor cells, highlight the complex roles platelets play in cancer biology. Therapeutic strategies aimed at inhibiting platelet activation have shown promising potential, with a variety of approaches being explored. Anti-platelet agents, such as aspirin and clopidogrel, as well as drugs that block key platelet receptors, thrombin activity, and platelet-derived microvesicles, are being investigated for their ability to reduce tumor progression and metastasis. Additionally, combining platelet-targeted therapies with conventional treatments, such as chemotherapy, immunotherapy, and anticoagulants, holds promise in enhancing the therapeutic outcomes for breast cancer patients. Nanoparticle-based therapies and gene silencing techniques represent innovative approaches for achieving more targeted and efficient treatments.

## Data Availability

Not applicable.
